# Interface Engineering of NCMA Cathodes with LATP Coatings for High-Performance Solid-State Lithium Batteries

**DOI:** 10.3390/nano15141057

**Published:** 2025-07-08

**Authors:** Shih-Ping Cho, Muhammad Usman Hameed, Chien-Te Hsieh, Wei-Ren Liu

**Affiliations:** 1 Department of Chemical Engineering, R&D Center for Membrane Technology, Chung Yuan Christian University, 200 Chung Pei Road, Chungli District, Taoyuan City 32023, Taiwan; ted10307@gmail.com; 2 Department of Chemistry, University of Poonch Rawalakot, Azad Kashmir 12350, Pakistan; drusmanhamid@upr.edu.pk; 3 Department of Chemical Engineering and Materials Science, Yuan Ze University, Taoyuan 32003, Taiwan; 4 Department of Mechanical, Aerospace, and Biomedical Engineering, University of Tennessee, Knoxville, TN 37996, USA; 5 Hierarchical Green-Energy Materials (Hi-GEM) Research Center, National Cheng Kung University, 1 University Road, Tainan 70101, Taiwan; 6 Department of Chemical Engineering, Faculty of Engineering, Chulalongkorn University, Bangkok 10330, Thailand

**Keywords:** NASICON-type phosphate coating, all-solid-state lithium batteries, sulfide electrolytes, interfacial stability, LiNi_0.83_Co_0.06_Mn_0.06_Al_0.05_O_2_

## Abstract

The development of high-performance and stable solid-state lithium batteries (SSBs) is critical for advancing next-generation energy storage technologies. This study investigates LATP (Li_1.3_Al_0.3_Ti_1.7_(PO_4_)_3_) coatings to enhance the electrochemical performance and interface stability of NCMA83 (LiNi_0.83_Co_0.06_Mn_0.06_Al_0.05_O_2_) cathodes. Compared to conventional combinations with LPSC (Li_6_PS_5_Cl) solid electrolytes, LATP coatings significantly reduce interfacial reactivity and improve cycling stability. Structural and morphological analyses reveal that LATP coatings maintain the crystallinity of NCMA83 while fine-tuning its lattice stress. Electrochemical testing demonstrates that LATP-modified samples (83L5) achieve superior capacity retention (65 mAh/g after 50 cycles) and reduced impedance (R_ct_ ~200 Ω), compared to unmodified samples (83L0). These results highlight LATP’s potential as a surface engineering solution to mitigate degradation effects, enhance ionic conductivity, and extend the lifespan of high-capacity SSBs.

## 1. Introduction

The development of high-performance, safe, and reliable solid-state batteries (SSBs) is critical for advancing next-generation energy storage technologies [1,2,3]. Among the promising candidates for cathode materials, nickel–cobalt–manganese LiNi_1−_*_x_*_−_*_y_*Co*_x_* Mn*_y_*O_2_ (NCM) stands out due to its high energy and power density [4]. When paired with solid-state electrolytes (SSEs) like Li-argyrodite (Li_6_PS_5_Cl, LPSC), SSBs can offer improved safety and ionic conductivity compared to conventional liquid electrolytes [5,6].

However, the degradation of NCM is a concern, prompting the use of NCMA as a replacement. The selection of LiNi_0.83_Co_0.06_Mn_0.06_Al_0.05_O_2_ (NCMA83) over LiNi_0.8_Co_0.1_Mn_0.1_O_2_ (NCM811) for battery cathodes is based on several factors, including bond dissociation energy (BDEO), cation mixing, and structural stability, which make NCMA83 more durable for high-performance applications. Specifically, the lower BDEO values for Ni^3+^ (390–450 kJ/mol) and Ni^2+^ (240–390 kJ/mol) in NCM811 lead to weaker Ni–O bonds, resulting in degradation. In contrast, Al^3+^ in NCMA83 has a higher BDEO (501 kJ/mol), providing enhanced stability. Furthermore, the presence of Al^3+^ in NCMA83 suppresses cation mixing, which helps maintain the layered structure and reduces microcracks—a common issue in high-nickel materials like NCM811. Al-doping stabilizes the lattice, minimizing abrupt structural changes during charging cycles, while maintaining a wider LiO_6_ interslab thickness in highly charged states for stable electrochemical function. Its rigid framework further boosts the cathode’s performance. This enhancement improves longevity and reduces impedance, yielding superior performance.

Nonetheless, significant challenges arise at the interface between the NCM cathode and the LPSC electrolyte, largely due to the limited electrochemical and chemical stability of LPSC [7,8]. While LPSC SSEs offer high ionic conductivity (σ = 1.33 × 10^−3^ S/cm at 25 °C), their narrow electrochemical stability window (ϕ relative to Li/Li^+^ at 1.7–2.5V) makes them susceptible to degradation when exposed to the higher voltages typical of NCM cathodes (typically 2.8–4.3V). This vulnerability leads to considerable instability and performance degradation over time. Additionally, LPSC’s poor chemical compatibility with NCM cathodes triggers undesirable side reactions, resulting in increased energy loss and reduced long-term stability. In visual representations of calculated reaction energies, the reaction energy for NCM811 paired with LPSC is notably high, around 500 meV/atom, confirming substantial interfacial instability. In contrast, NCM811 with LATP (Li_1.3_Al_0.3_Ti_1.7_(PO_4_)_3_) exhibits significantly lower reaction energy, approximately 100 meV/atom, suggesting a much more stable interface and reduced energy loss compared to the LPSC combination [9,10,11,12].

To address these interfacial challenges, surface engineering techniques have emerged as vital strategies for stabilizing the interface between electrodes and electrolytes in advanced battery technologies [13,14]. These methods aim to mitigate interfacial reactivity, enhance cycling stability, and improve overall battery performance. Key strategies include doping [15]. and applying various coatings [16,17,18,19,20,21,22,23,24,25,26,27,28,29,30,31,32]. Doping introduces elements like aluminum (Al), titanium (Ti), and niobium (Nb) to decrease interfacial reactivity between electrodes and solid electrolytes, thus minimizing unwanted side reactions and enhancing structural stability [15]. Coating electrodes with protective layers improves interfacial stability. Various materials explored include the following: Metal oxides: Coatings like LiAlO_2_ and Li_4_Ti_5_O_12_ (LTO) have shown significant benefits. For example, a LiAlO_2_-coated Li(Ni_1/3_Mn_1/3_Co_1/3_)O_2_ cathode achieved an initial discharge capacity of 134 mAh/g, retaining over 124 mAh/g after 400 cycles. LTO coatings improved cycling performance, with a capacity retention of 48% after 100 cycles. Silica (SiO_2_): SiO_2_-coated lithium-rich layered oxides achieved an initial capacity of 212 mAh/g, with improved thermal stability. Phosphates: Li_3_PO_4_ (LPO) coatings enhanced lithium-ion diffusion and cycling stability, achieving a capacity of 177 mAh/g. Boron compounds: Lithium borate coatings improved cycling stability, achieving a specific capacity of 75 mAh/g. Halide coatings: Li_2_ZrCl_6_ halide layers reduced interfacial resistance, retaining 91.2% capacity after 100 cycles. Polymer coatings: Polyacrylonitrile (PAN) coatings improved performance, with Ni-rich NMC811 cathodes achieving an initial capacity of 192.16 mAh/g [16,17,18,19,20,21,22,23,24,25,26,27,28,29,30,31,32].

Recent studies support these approaches. Wu et al. demonstrated that an LATP coating significantly enhances the electrochemical performance of high-nickel LiNi_0.83_Co0._11_Mn_0.06_O_2_ (NCM83) cathodes, achieving a discharge capacity of 179.3 mAh/g and a capacity retention of 69.67% after 300 cycles [33]. Shao et al. showed that LATP coatings stabilize NCM811, enhancing their performance in lithium-ion and solid-state batteries while delaying exothermic reactions [34]. Zhang et al. found that LATP coating on LiNi_1/3_Co_1/3_Mn_1/3_O_2_ (NCM111) improved discharge capacity retention to 92.37% after 100 cycles at a high cut-off voltage of 4.6 V, suggesting LATP’s effectiveness in enhancing cycle stability in high-capacity cathode materials [35].

We use LATP as a buffer layer coating to modify polycrystalline NCMA83 cathodes for all solid-state lithium-ion batteries. LATP is a highly ionic conductor with an excellent Li^+^ transport rate of 7 × 10^−4^ S/cm [36]. It offers several advantages, including high ionic conductivity and superior chemical stability, making it compatible with both NCM and LPSC [37,38]. Moreover, LATP has a wider electrochemical stability window (ϕ relative to Li/Li^+^ at 2.17–4.21 V), which reduces the risk of interface degradation when in contact with NCM. By applying LATP as a coating, we aim to address the challenges posed by the NCMA-LPSC interface, enhancing electrochemical performance and extending battery life. This work investigates the effectiveness of LATP as a surface engineering solution for NCMA83 cathodes, focusing on its ability to mitigate interface issues while preserving the high performance of sulfide solid-state batteries [39,40,41].

## 2. Materials and Methods

### 2.1. Materials and Chemicals

This study focuses on materials utilized for the preparation of LATP (lithium aluminum titanium phosphate) coatings on NCMA83 cathodes, a high-performance lithium-ion battery material. The cathode (NCMA83) composition, Li_1.01_Ni_0.83_Co_0.11_Mn_0.05_Al_0.01_O_2,_ is supplied by Aleees Electric Co., Ltd., Taoyuan, Taiwan. Key LATP precursors include aluminum nitrate (Al(NO_3_)_3_·9H_2_O) with 98% purity, sourced from Luxion Limited, and lithium hydroxide (LiOH·H_2_O) of identical purity, provided by Acros Organics B.V. Titanium isopropoxide (Ti(OCH(CH_3_)_2_)_4_), a critical component for LATP synthesis, is supplied by Thermo Scientific Chemicals with 98% purity. Phosphoric acid (H_3_PO_4_), at 86% purity, is obtained from Sigma-Aldrich, while 95% pure ethanol (C_2_H_5_OH), used as a solvent, is acquired from Jingming Chemical Co., Ltd., Chongqing, China. These high-purity materials ensure the integrity of the LATP coating process, which aims to enhance the stability and performance of NCMA83 cathodes in lithium-ion batteries.

### 2.2. Synthesis of NCMA83@LATP-Coated Cathodes

**Scheme 1:**
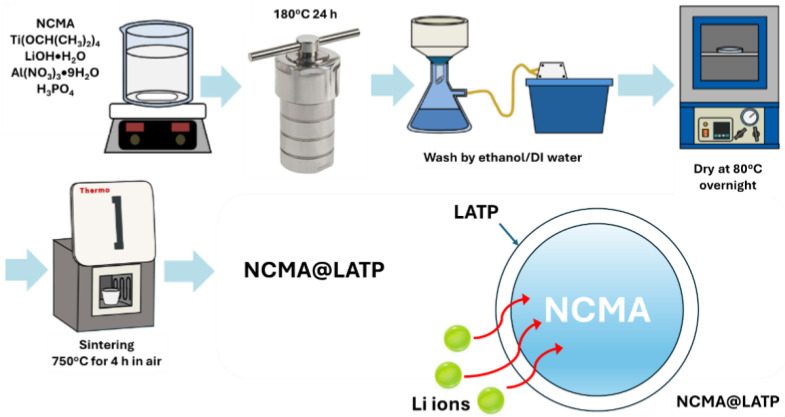
Synthesis of NASICON-type phosphate coating Li_1.3_Al_0.3_Ti_1.7_(PO_4_)_3_ on the NCMA cathode using hydrothermal methods.

The precursors, namely Ti(OCH(CH_3_)_2_), Al(NO_3_)_3_·9H_2_O, H_3_PO_4_, and LiOH·H_2_O, were mixed in a ratio of Li:Al:Ti:P mol% **=** 1.3:0.3:1.7:3 into a beaker, as shown in Scheme 1. Then, alcohol was added, and the mixture was stirred for more than an hour to ensure dissolution and the formation of an LATP precursor solution. Subsequently, NCMA83 material should be added to the precursor solution by weight, after which the solution should be transferred to a 100 mL Teflon autoclave. The autoclave should then be heated in an oven to 180 °C for 24 h to facilitate a hydrothermal reaction. The hydrothermal solution should then be filtered under reduced pressure to obtain an off-white precipitate, which should then be dried overnight at 80 °C in an oven to produce the hydrothermal precursor. This precursor was subsequently heated in air at 750 °C for 4 h and allowed to cool naturally to room temperature, resulting in the NCMA@LATP% powder. Different weight percentages of LATP were coated with NCMA83@LATP 1% to create samples labeled as bare (83L0), 83L1, 83L3, and 83L5. For instance, NCMA83@LATP 1% is denoted as 83L1. Further studies will follow.

### 2.3. Material Characterizations

The crystallographic phase was identified by X-ray powder diffraction (XRD) on a Bruker eco D8 advance diffractometer using monochromatic CuKα radiation (λ = 1.54060Å). The samples were analyzed using closed XRD plates, and the XRD data were collected at a scan rate of 2° min^−1^ in steps of 0.02° in the two ranges of 10–80°. Rietveld structure refinement from the XRD data was performed using TOPAS software (ver. 6.0.0.9). The crystal structure was determined using the Diamond Crystal and Molecular Structure (ver. 3.2) Visualization software. Quantitative elemental analyses and elemental distributions were obtained using energy dispersive spectrometry and electron mapping, while images of the morphology and microstructure of the materials were obtained using field emission scanning electron microscopy (FE-SEM, JSM-7600F; JEOL) (EDS, X-MAX, Oxford instrument). X-ray photoelectron spectroscopy (XPS) was used to determine the chemical valence states using a Kα XPS spectrometer (Thermo Scientific). In addition, energy dispersive spectroscopy (EDS) was used for elemental mapping analysis of the sample. In this study, the electrochemical performance of lithium-ion coin cells and sulfide solid-state batteries (SSBs) was evaluated under varying conditions. In parallel, sulfide SSBs were fabricated using NCMA83 as the cathode, Li6PS5Cl as the solid electrolyte, and a lithium-indium (LiIn) alloy foil as the anode. The solid electrolyte was compressed into a 10 mm pellet, and a composite cathode comprising NCMA83 and Li_6_PS_5_Cl in a 70:30 weight ratio was pressed onto one side of the pellet with a loading of ~8 mg cm^−2^. The LiIn alloy foil was attached to the opposite side, and the assembly was compressed under 80 MPa with stainless steel as the current collector. Figure S1 shows the stack cell composition and pressure recorder. Figure S2 illustrates both sides of the cell pellet following cell assembly. Electrochemical performance was evaluated in a Swagelok cell configuration at 55 °C, with charge–discharge cycling conducted over a voltage range of 2.7–4.3 V (vs. InLi^+^/Li) at a rate of 0.1 C.

### 2.4. Electrochemical Characterization

Using an E.C. Lab Biologic Potentiostat, model SP-200, experiments were performed to measure the samples impedance in the frequency range of 7–10 MHz at an A.C. amplitude of 10 mV using electrochemical impedance spectroscopy (EIS). The as-obtained impedance values and phase angles were converted to real and imaginary parts of the capacitance impedance using the EC-Lab program, which enabled Nyquist plots to be drawn.

### 2.5. Cell Assembly for Lithium-Ion Battery Testing

In this study, the electrochemical performance of lithium-ion coin cells and sulfide solid-state batteries (SSBs) was evaluated under varying conditions. In parallel, sulfide SSBs were fabricated using NCMA83 as the cathode, Li_6_PS_5_Cl as the solid electrolyte, and a lithium-indium (LiIn) alloy foil as the anode. The solid electrolyte was compressed into a 10 mm pellet, and a composite cathode comprising NCMA83 and Li_6_PS_5_Cl in a 70:30 weight ratio was pressed onto one side of the pellet with a loading of ~8 mg cm^−2^. The LiIn alloy foil was attached to the opposite side, and the assembly was compressed under 80 MPa with stainless steel as the current collector. Electrochemical performance was evaluated in a Swagelok cell configuration at 55 °C, with charge–discharge cycling conducted over a voltage range of 2.7–4.3 V (vs. InLi^+^/Li) at a rate of 0.1 C.

## 3. Results and Discussion

### 3.1. Structure and Morphology

**Figure 1:**
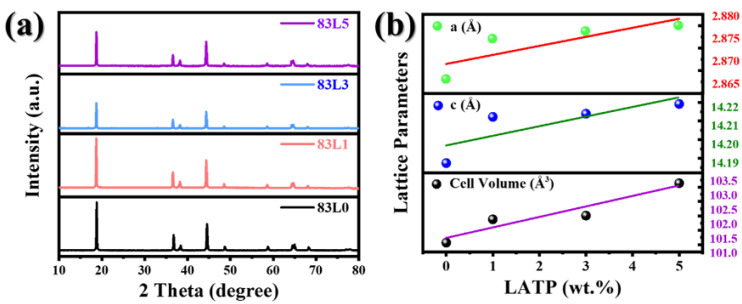
(**a**) XRD patterns of pristine and LATP-coated NCMA83; (**b**) unit cell parameters of different coating ratios (0%, 1%, 3%, 5% wt.).

Figure 1a,b show the XRD patterns of the samples with different coating ratios (1%, 3%, 5% wt.) and the trend of changes in the refined unit cell parameters *a*, *c* and unit cell volume. In Figure 1a, the XRD pattern showed that the main phase of all samples remained the same, and no new phase or significant change in crystal structure occurred with the increase in coating ratio, indicating that the coating process does not destroy the crystallinity of cathode materials. In addition, as the LATP coating ratio increased, the position of the main diffraction peaks in the XRD pattern remained almost unchanged, with only slight changes in peak shape and peak intensity. This may be related to the effect of the coating layer on the crystal surface or local structure.

Figure 1b further analyzed the changes in the unit cell parameters *a* and *c* and the unit cell volume through XRD refinement. The results showed that with the increase in the coating content, the unit cell parameters a and c both showed a slight increasing trend, and the unit cell volume also gradually increased. These changes may be due to the introduction of the coating layer, which fine-tunes the lattice stress or changes the local crystal structure. It was worth noting that the increase in the unit cell volume was relatively small, indicating that the increase in the coating ratio has a limited effect on the structure of the material’s main phase and does not lead to significant changes in the crystal structure.

**Figure 2:**
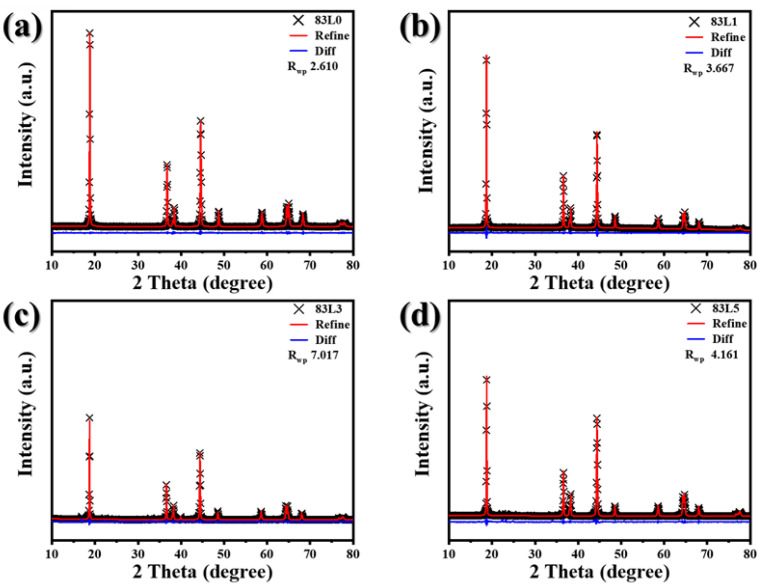
XRD patterns and corresponding Rietveld refinement results of as-prepared (**a**) 83L0, (**b**) 83L1, (**c**) 83L3, and (**d**) 83L5.

Figure 2a–d show the XRD patterns and their refinement results for samples 83L0, 83L1, 83L3, and 83L5, respectively, further demonstrating the characteristics of the crystal structure at different coating ratios. It can be seen from these patterns that as the coating ratio increases, the position of the main diffraction peak of the sample remains stable, but changes in the shape and intensity of the peak may reflect the slight effect of the coating layer on the crystal surface or internal structure. These results are consistent with the trend of changes in the cell parameters and cell volume in Figure 1b, indicating that the increase in the coating ratio mainly affects the crystal structure through subtle structural adjustments rather than significant structural damage.

Table 1 systematically compiles the results of the XRD refinement, including unit cell parameters a and c and the unit cell volume, and corresponds to the graphical results in Figure 1b. It can be intuitively seen from the table that as the coating ratio increases from 1 wt.% to 5 wt.%, the values of the unit cell parameters and the unit cell volume show a gradual increasing trend. This further confirms the trend of the effect of the coating ratio on the crystal structure and provides quantitative support for the changes in peak shape and peak intensity observed in Figure 1a and Figure 2. An increase in the coating ratio does not lead to a change in the main phase of the material, and the crystallographic structure of the sample remains stable. With the increase in the coating ratio, the unit cell parameters a and c and the unit cell volume all showed a gradual increasing trend, indicating that the introduction of the coating layer has a slight adjustment effect on the crystal structure. These small structural changes show that the regulation of the coating ratio has good controllability over the structural stability of the material.

The thickness of the LATP coater was too thin to identify the structure. The LATP thickness was less than 8 nm, whereas the NCMA particle radius exceeds 4 μm. This indicated that the concentration of the LATP coatings compared to the NCMA was quite minimal. However, as shown in Figure S3, the amount of LATP was reported to be 100%. This disparity in proportions could be a critical factor influencing the results. Therefore, LATP powders were synthesized using the NCMA83@LATP-coated cathodes procedure without adding NCMA. Figure S3 displays the XRD pattern of the LATP-like coater. Additionally, EIS measurements were conducted to calculate the ionic conductivity of the coater, which was found to be 1.21 × 10^−5^ S/cm, lower than the common LATP SSE by nearly one degree in value (10^−4^), as shown in Table S1. These data support the conclusion that Li-ion transport occurs through the LATP coater.

**Figure 3:**
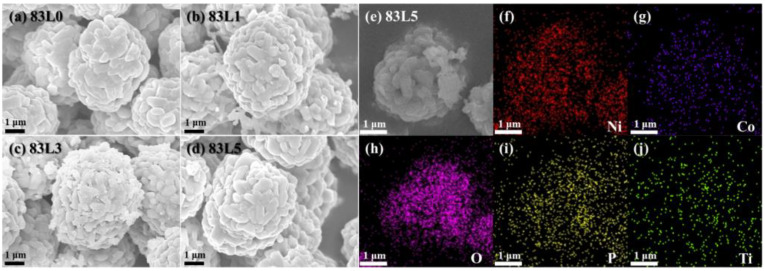
SEM images of (**a**) 83L0, (**b**) 83L1, (**c**) 83L3, (**d**) 83L5, and the corresponding EDS mappings for (**e**) 83L5 in (**f**) Ni, (**g**) Co, (**h**) O, (**i**) P and (**j**) Ti element.

**Figure 4:**
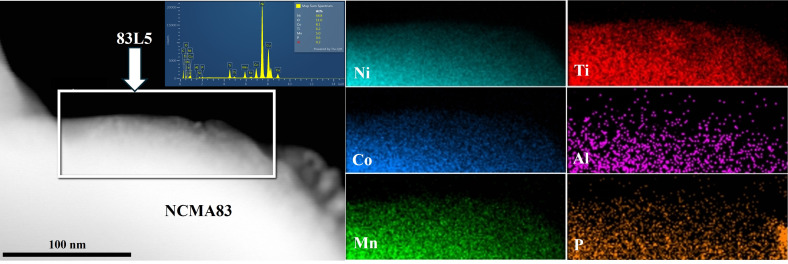
TEM and EDS images of 83L5, Ni, Ti, Co, Al, Mn, and P elemental mapping.

Based on the SEM, TEM, and EDS analysis results from Figure 3 and Figure 4, the results demonstrated the effects of LATP coating on NCMA83 cathode materials at varying coating ratios (0%, 1%, 3%, and 5% wt.). SEM images reveal that the NCMA particles maintain their spherical morphology with diameters ranging from 3.85 to 4.12 μm, indicating that the LATP coating process does not compromise the structural integrity of the cathode material. As the coating ratio increases, the particle surfaces become progressively smoother, with the 5% wt. coating (83L5) forming a uniform and dense layer. EDS mapping further confirms the successful formation of LATP coatings, as evidenced by the reduced intensity of Ni and Co signals (from the NCMA substrate) and the strong, uniform distribution of LATP-specific elements (Ti, P, Al). In particular, the 5% wt. coating demonstrates complete coverage, effectively reducing substrate exposure and providing robust surface protection. Overall, this study highlights the potential of LATP coatings in advancing NCMA cathode material performance without compromising its structural properties.

**Figure 5:**
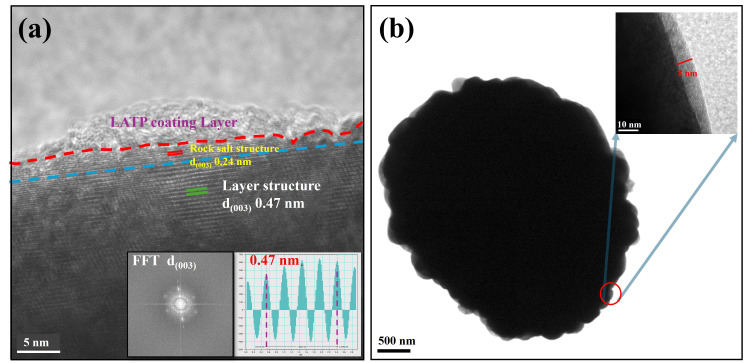
TEM Images of 83L5 (**a**) d-spacing, FFT and (**b**) thickness of the LATP coating layer.

The analysis of Figure 5 underscores the structural and compositional variations between the surface and bulk regions of NCM (Ni-Co-Mn cathode material) and the LATP coating layer, approximately 8–10 nm thick, using high-resolution transmission electron microscopy (HRTEM). The TEM images revealed a Ni-rich rock-salt phase at the surface, contrasting with the layered structure observed in the bulk, as evidenced by the (003) planes characteristic of layered materials. A red line near the surface (~10 nm) corresponds to the rock-salt phase, while Co and Mn concentrations stabilize deeper into the material, aligning with the uniform layered structure in the bulk. High-resolution HRTEM further confirms this structural transition: the surface region exhibits a disordered Ni-rich rock-salt phase with an interatomic distance of d = 2.4 Å, while the bulk maintains an ordered layered configuration with an interatomic distance of d = 4.7 Å. Atomic models illustrate these differences, showing that in the rock-salt phase, transition metals (Ni, Co, Mn) occupy octahedral sites and lithium ions are in tetrahedral sites. In contrast, the layered structure features lithium ions situated between TM layers in an R-3m symmetry, preserving the desirable layered arrangement. The formation of the rock-salt phase at the surface is attributed to transition metal oxidation and lithium loss, common degradation mechanisms in NCMA materials. This transformation is critical as the rock-salt phase increases interfacial impedance, impeding lithium-ion transport and degrading electrochemical performance. These findings highlight the importance of surface engineering strategies, such as LATP coatings, to mitigate degradation effects and enhance material stability.

The sintering process between NCMA83 cathodes and LATP precursors poses challenges due to inherent chemical instability. While NCMA83 initially retains a stable layered structure, exposure to elevated temperatures (750°C for 4 h) triggers the migration of Li-ions from the NCMA83 bulk to the NCMA83/LATP interface. This migration results in the formation of Li_3_PO_4_. As Li-ion migration progresses, Ni^2+^ ions gradually occupy Li sites owing to their comparable ionic radii (Ni^2+^: 0.69 Å, Li^+^: 0.76 Å), causing severe cation mixing. This phenomenon inhibited Li-ion mobility and decreased the cathode’s capacity. In addition, side reactions between NCMA83 and LATP intensify the degradation of NCMA83’s layered structure, irreversibly transforming it into a spinel phase. Data presented in Table 1 demonstrate that the I(003)/I(104) ratio for samples 83L0, 83L1, 83L3, and 83L5 progressively decreases with an increase in LATP layer count. This decline reflects heightened Ni^2+^/Li^+^ cation mixing, likely driven by phase transitions resulting from Li/Ni intermixing during sintering. Additionally, Figure 5a reveals the formation of rock-salt structures at the NCMA83-LATP interface. These findings highlight the critical impact of Ni^2+^-induced cation mixing and the side reactions occurring during sintering.

**Figure 6:**
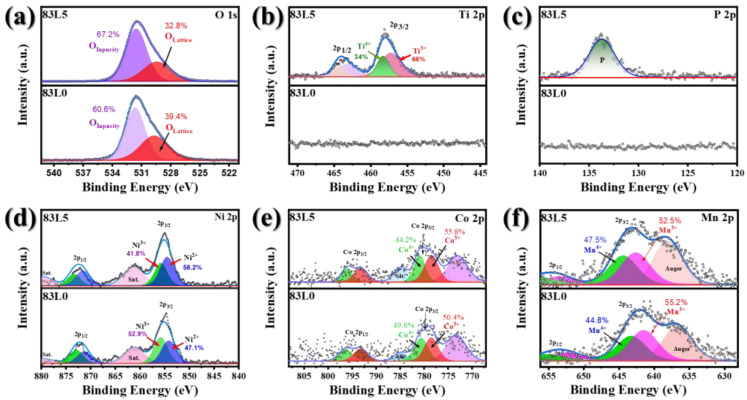
XPS spectra of comparison 83L5 and 83L0 in O 1s (**a**), Ti 2p (**b**), P 2p (**c**), Ni 2p (**d**), Co 2p (**e**), and Mn 2p (**f**) binding energies.

Based on the XPS analysis, this study investigated the changes in surface elemental states of the samples, leading to the following observations and conclusions. The XPS spectra in Figure 6 indicate consistent peak patterns before and after coating, suggesting that the bulk structure of the samples remains largely unchanged. Notably, Figure 6b reveals distinct Ti 2p peaks on the surface of NCMA83 after LATP modification, which were absent in the uncoated sample, confirming that Ti originates from LATP and was localized on the modified surface. Furthermore, Figure 6a–f illustrate the XPS results for O 1s, Ti 2p, P 2p, Ni 2p, Co 2p, and Mn 2p. The significant signals of Ti and P confirmed the successful deposition of LATP on the NCMA83 surface. Meanwhile, the minimal changes in Ni, Co, and Mn peaks indicate that the surface chemical states of these metallic elements remain stable after coating. In summary, XPS analysis demonstrated that LATP modification effectively introduces Ti and P elements onto the NCMA83 surface without altering its bulk structure or the surface valence states of other metals, highlighting the stability and efficacy of this surface modification technique.

**Figure 7:**
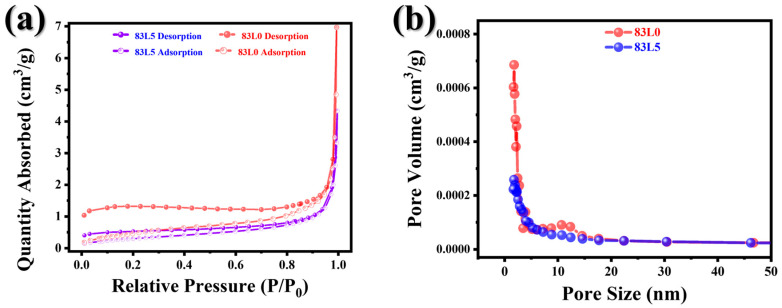
(**a**) N_2_ adsorption/desorption isotherms and (**b**) pore sizes of 83L0 and 83L5.

As demonstrated in Figure 7, N_2_ adsorption/desorption isotherms exhibited a comparable morphology for both samples. However, it is observed that the endpoint of the desorption curve is unable to align with the initial point of the adsorption curve for both samples. This discrepancy can be attributed to the comparatively diminished surface area of the samples. Utilizing the data presented in Table 2, the surface areas of 83L0 and 83L5 are estimated to be approximately 1.649 m^2^ g^−1^ and 1.077 m^2^ g^−1^, respectively. Furthermore, the pore sizes of 83L0 and 83L5 are determined to be 16.526 and 18.103 nm, respectively.

The comparative analysis of uncoated (83L0) and 5% LATP-coated (83L5) cathodes reveals the significant impact of surface modification on electrochemical performance. The LATP coating fundamentally alters the reaction kinetics from a diffusion-controlled mechanism (83L0) to a more surface-controlled process (83L5), as evidenced by the decrease in *b*-values from ~0.5 to ~0.3. While the coating reduces the diffusion coefficient by an order of magnitude from 10^−6^ to 10^−7^ cm^2^/s, it provides crucial benefits in terms of cycling stability and electrode reversibility.

### 3.2. Electrochemical Analysis

**Figure 8:**
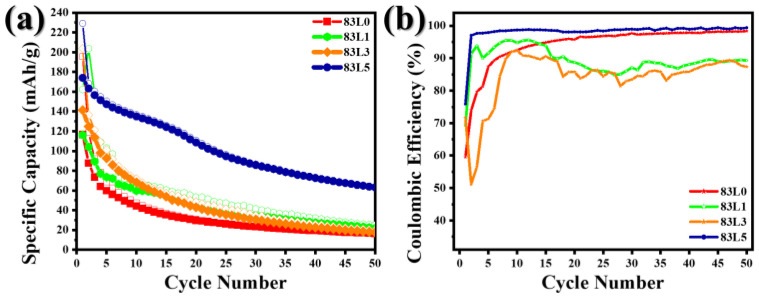
Comparison of different LATP weight ratios of (**a**) cyclic charge–discharge capacity performance and (**b**) coulombic efficiency in the voltage range of 2.7–4.3V at 0.1C under 55 °C.

**Figure 9:**
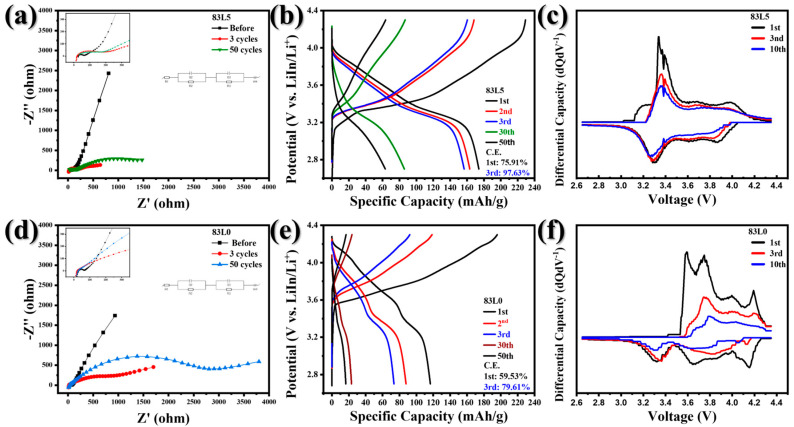
Comparison of electrochemical performance of the 83L5 and 83L0 cathodes in LPSC SSE with LiIn metal anode. (**a**,**d**) EIS results before and after the 3rd and 50th cycles; (**b**,**e**) the charge–discharge curves after the 1st, 2nd, and 50th cycles; (**c**,**f**) the dQ/dV profiles after the 1st, 3rd, and 10th cycles in the voltage range of 2.7–4.3V at 0.1C at 55 °C.

Based on the data and analysis from Figure 8 and Figure 9, the results highlight the significant advantages of LATP modification in enhancing the electrochemical performance of NCMA83 electrode materials in the voltage range of 2.7–4.3V at 0.1C under 55 °C. From Figure 8a,b, after 50 charge–discharge cycles, the LATP-modified sample, 83L5, retained a capacity of 65 mAh/g with a stable Coulombic efficiency of 98–99.5%, demonstrating superior stability and performance compared to the unmodified 83L0, which showed a drastic capacity drop to 18 mAh/g. This indicates that LATP modification effectively improves capacity retention and stability. Figure 9 further supports these findings through EIS, charge–discharge, and dQ/dV analyses. The EIS results reveal that 83L5 exhibited a significantly lower charge transfer resistance (R_ct_) of approximately 200 Ω after 50 cycles, compared to 2000 Ω for 83L0, indicating smoother ion transport due to improved interfacial properties. The charge–discharge curves confirmed higher specific capacity for 83L5 (65 mAh/g) versus 83L0 (18 mAh/g), underscoring enhanced electrochemical activity. Additionally, the dQ/dV analysis showed that 83L5 has minimal polarization (0.01 V) relative to the severe polarization of 83L0 (0.3 V), reflecting faster and more stable electrochemical reactions in the modified material. Collectively, these results demonstrated that LATP modification significantly improved the interfacial characteristics, stability, and overall electrochemical performance of NCMA83, while unmodified 83L0 suffers from poor stability, high impedance, and severe polarization. This underscores the critical role of LATP modification in advancing electrode material performance.

The HRTEM image revealed that the coating layer exhibited inconsistencies in thickness. Additionally, the NCMA cathode was prone to approximately 5% volume expansion and contraction during charge and discharge cycles. This repeated stress induced cracking in thinner regions and boundary areas of the coating. As a result, the exposed NCMA reacted with sulfide electrolytes, causing a significant reduction in specific capacity after 10 cycles.

**Figure 10:**
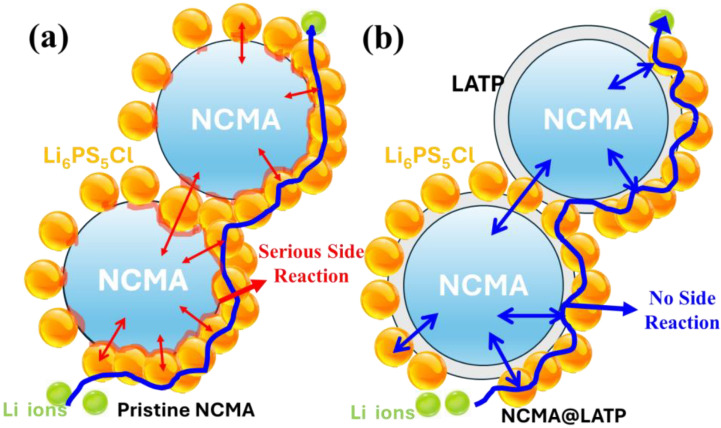
Scheme of fading mechanism: (**a**) bare; (**b**) coated NCMA83.

The electrochemical characterization through GITT (Figure S5) and CV analyses (Figures S6 and S7) demonstrated that the LATP coating acts as a protective layer, moderating lithium-ion transport while enhancing the overall electrode stability. This trade-off between diffusion rate and stability suggested, as shown in Figure 10, that optimal coating thickness was crucial for balancing performance parameters. The findings indicated that surface modification with LATP was a promising strategy for improving the long-term performance and reliability of cathode materials in lithium-ion batteries.

## 4. Conclusions

This study demonstrates the effectiveness of LATP coatings in improving the stability and performance of NCMA83 cathodes for solid-state lithium batteries. Key findings include the following: LATP coatings provide a wider electrochemical stability window and higher ionic conductivity, effectively addressing the interfacial instability and reactivity issues between NCMA and LPSC. Structural analyses confirm that LATP coatings do not alter the primary crystal structure of NCMA83 but contribute to subtle lattice adjustments, enhancing material robustness. Electrochemical tests show that LATP-modified cathodes (83L5) achieve superior capacity retention, reduced polarization, and lower interfacial resistance compared to unmodified samples (83L0). In conclusion, LATP coatings present a promising surface engineering approach to overcome the limitations of high-nickel cathode materials in solid-state battery applications, paving the way for more durable and high-performance energy storage systems. Future work will focus on optimizing coating processes and exploring additional solid-state electrolyte combinations to further enhance battery performance.

## Figures and Tables

**Table 1:** Lattice parameters, cell volume, R_wp_, and I_(003)_/I_(104)_ ratios of 83L0, 83L1, 83L3, and 83L5.

Samples	*a* (Å)	*c* (Å)	Cell Volume (Å^3^)	R_wp_ (%)	I(003)/I(104)
83L0	2.86810(3)	14.1874(7)	101.069(9)	2.610	1.78513
83L1	2.87706(5)	14.2122(4)	101.881(5)	3.666	1.76049
83L3	2.87873(4)	14.2139(7)	102.011(4)	7.017	1.51346
83L5	2.87999(1)	14.2191(7)	103.137(9)	4.161	1.37251

**Table 2:** BET characterization results of 83L0 and 83L5.

Samples	Surface Area (m^2^/g)	Pore Volume (cm^3^/g)	Pore Size (nm)
83L0	1.6499	0.007480	16.52651
83L5	1.0773	0.005147	18.10328

## Data Availability

The original contributions presented in this study are included in the article/Supplementary Material. Further inquiries can be directed to the corresponding authors.

## Supplementary Materials

The following supporting information can be downloaded at: https://www.mdpi.com/article/10.3390/nano15141057/s1. Figure S1: (a,c) stack cell; (b) composition of all solid-state electrolyte battery. Figure S2: (a) Cathode side of cell pellet; (b) LiIn metal anode side of cell pellet. Figure S3. XRD patterns of as-synthesized LATP (namely Wet LATP) and its standard pattern taken from ICSD 14585. Figure S4. EIS of as-synthesized LATP. Figure S5. (a–d): GITT for pristine cathode and 5%LATP coated cathode. Figure S6. L0 (Pristine cathode) CV (a) the first 3 cycles, (b) different scan V; (c) the b value, and (d) D value. Figure S7. L5 (5% LATP-coated cathode) CV (a) the first 3 cycles, (b) different scan V; (c) the b value, and (d) D value. Figure S8. SEM images of (a) 83L0 and (b) 83L5 composite cathode after 50 charge-discharge cycles. Table S1. Ionic conductivity of LATP.

## References

[1] Xu J.J., Cai X.Y., Cai S.M., Shao Y.X., Hu C., Lu S.R., Ding S.J. (2023). High-Energy Lithium-Ion Batteries: Recent Progress and a Promising Future in Applications. Energy Environ. Mater..

[2] Wu D.X., Wu F. (2023). Toward better batteries: Solid-state battery roadmap 2035+. Etransportation.

[3] Schmaltz T., Hartmann F., Wicke T., Weymann L., Neef C., Janek J. (2023). A Roadmap for Solid-State Batteries. Adv. Energy Mater..

[4] Liu W., Li D., Liu Y., Luo D., Xu R. (2024). A Critical Review of Single-Crystal LiNi*_x_*Mn*_y_*Co_1-_*_x_*_-_*_y_*O_2_ Cathode Materials. Renewables.

[5] Bai X.T., Duan Y., Zhuang W.D., Yang R., Wang J.T. (2020). Research progress in Li-argyrodite-based solid-state electrolytes. J. Mater. Chem. A.

[6] Yu C., Zhao F.P., Luo J., Zhang L., Sun X.L. (2021). Recent development of lithium argyrodite solid-state electrolytes for solid-state batteries: Synthesis, structure, stability and dynamics. Nano Energy.

[7] Zhu Y.Z., He X.F., Mo Y.F. (2016). First principles study on electrochemical and chemical stability of solid electrolyte-electrode interfaces in all-solid-state Li-ion batteries. J. Mater. Chem. A.

[8] Cho H., Kim J., Kim M., An H., Min K., Park K. (2024). A review of problems and solutions in Ni-rich cathode-based Li-ion batteries from two research aspects: Experimental studies and computational insights. J. Power Sources.

[9] Auvergniot J., Cassel A., Ledeuil J.B., Viallet V., Seznec V., Dedryvère R. (2017). Interface Stability of Argyrodite Li_6_PS_5_Cl toward LiCoO_2_, LiNi_1/3_Co_1/3_Mn_1/3_O_2_, and LiMn_2_O_4_ in Bulk All-Solid-State Batteries. Chem. Mater..

[10] Zhu Y., He X., Mo Y. (2015). Origin of Outstanding Stability in the Lithium Solid Electrolyte Materials: Insights from Thermodynamic Analyses Based on First-Principles Calculations. ACS Appl. Mater. Interfaces.

[11] Chen Y., Huang L., Zhou D.L., Gao X., Hu T.F., Zhang Z.Y., Zhen Z., Chen X.D., Cui L.F., Wang G.X. (2024). Elucidating and Minimizing the Space-Charge Layer Effect between NCM Cathode and Li_6_PS_5_Cl for Sulfide-Based Solid-State Lithium Batteries. Adv. Energy Mater..

[12] Zhou L., Minafra N., Zeier W.G., Nazar L.F. (2021). Innovative Approaches to Li-Argyrodite Solid Electrolytes for All-Solid-State Lithium Batteries. Acc. Chem. Res..

[13] Li N.A., Luo J.Y., Zhu J.H., Zhuang X.D. (2023). Cathodic interface in sulfide-based all-solid-state lithium batteries. Energy Storage Mater..

[14] Meng J.K., Qu G., Huang Y.H. (2023). Stabilization strategies for high-capacity NCM materials targeting for safety and durability improvements. Etransportation.

[15] Sun H.H., Kim U.H., Park J.H., Park S.W., Seo D.H., Heller A., Mullins C.B., Yoon C.S., Sun Y.K. (2021). Transition metal-doped Ni-rich layered cathode materials for durable Li-ion batteries. Nat. Commun..

[16] Morchhale A., Tang Z.H., Yu C.Y., Farahati R., Kim J.H. (2023). Coating materials and processes for cathodes in sulfide-based all solid-state batteries. Curr. Opin. Electrochem..

[17] Okada K., Machida N., Naito M., Shigematsu T., Ito S., Fujiki S., Nakano M., Aihara Y. (2014). Preparation and electrochemical properties of LiAlO_2_-coated Li(Ni_1/3_Mn_1/3_Co_1/3_)O_2_ for all-solid-state batteries. Solid State Ion..

[18] Negi R.S., Minnmann P., Pan R.J., Ahmed S., Herzog M.J., Volz K., Takata R., Schmidt F., Janek J., Elm M.T. (2021). Stabilizing the Cathode/Electrolyte Interface Using a Dry-Processed Lithium Titanate Coating for All-Solid-State Batteries. Chem. Mater..

[19] Strauss F., Teo J.H., Maibach J., Kim A.Y., Mazilkin A., Janek J., Brezesinski T. (2020). Li_2_ZrO_3_-Coated NCM622 for Application in Inorganic Solid-State Batteries: Role of Surface Carbonates in the Cycling Performance. ACS Appl. Mater. Interfaces.

[20] James Abraham J., Nisar U., Monawwar H., Abdul Quddus A., Shakoor R.A., Saleh M.I., Kahraman R., Al-Qaradawi S., Aljaber A.S. (2020). Improved electrochemical performance of SiO_2_-coated Li-rich layered oxides-Li_1.2_Ni_0.13_Mn_0.54_Co_0.13_O_2_. J. Mater. Sci. Mater. Electron..

[21] Li X., Jin L., Song D., Zhang H., Shi X., Wang Z., Zhang L., Zhu L. (2020). LiNbO_3_-coated LiNi_0.8_Co_0.1_Mn_0.1_O_2_ cathode with high discharge capacity and rate performance for all-solid-state lithium battery. J. Energy Chem..

[22] You M.J., Jung J., Byeon Y.S., Jung J.Y., Hong Y., Park M.S. (2024). Controlled crystallinity of LiTaO_3_ surface layer for single-crystalline Ni-rich cathodes for lithium-ion batteries and all-solid-state batteries. Chem. Eng. J..

[23] Su Y., Chen G., Chen L., Shi Q., Lv Z., Lu Y., Bao L., Li N., Chen S., Wu F. (2021). Roles of Fast-Ion Conductor LiTaO_3_ Modifying Ni-rich Cathode Material for Li-Ion Batteries. ChemSusChem.

[24] Zhong Y., Fan Z., Zhang D., Su M., Wang X., Tu J. (2023). Surface Construction of a High-Ionic-Conductivity Buffering Layer on a LiNi_0.6_Co_0.2_Mn_0.2_O_2_ Cathode for Stable All-Solid-State Sulfide-Based Batteries. J. Electron. Mater..

[25] Zhou X., Deng L.H., Zhang K., Zhang Z.Y., Zhang L.L., Li Z., Kong T.Y., Xie Y.H., Wang Y.G. (2024). High-Performance Sulfide All-Solid-State Batteries Enabled by High-Voltage Ni-Rich Cathode with a Conformal and Conductive Protective Layer. ACS Appl. Energy Mater..

[26] Wu E.A., Jo C., Tan D.H.S., Zhang M.H., Doux J.M., Chen Y.T., Deysher G., Meng Y.S. (2020). A Facile, Dry-Processed Lithium Borate-Based Cathode Coating for Improved All-Solid-State Battery Performance. J. Electrochem. Soc..

[27] Shi J., Ma Z., Wu D., Yu Y., Wang Z., Fang Y., Chen D., Shang S., Qu X., Li P. (2024). Low-cost BPO_4_ In Situ Synthetic Li_3_PO_4_ Coating and B/P-Doping to Boost 4.8 V Cyclability for Sulfide-Based All-Solid-State Lithium Batteries. Small.

[28] Zhang A., Wang J., Yu R., Zhuo H., Wang C., Ren Z., Wang J. (2023). Practical Application of Li-Rich Materials in Halide All-Solid-State Batteries and Interfacial Reactions between Cathodes and Electrolytes. ACS Appl. Mater. Interfaces.

[29] Cha H., Yun J., Kim S., Kang J., Cho M., Cho W., Lee J.-W. (2024). Stabilizing the interface between high-Ni oxide cathode and Li_6_PS_5_Cl for all-solid-state batteries via dual-compatible halides. J. Power Sources.

[30] Lee D., Cui Z., Goodenough J.B., Manthiram A. (2024). Interphase Stabilization of LiNi_0.5_Mn_1.5_O_4_ Cathode for 5 V-Class All-Solid-State Batteries. Small.

[31] Demuth T., Fuchs T., Walther F., Pokle A., Ahmed S., Malaki M., Beyer A., Janek J., Volz K. (2023). Influence of the sintering temperature on LLZO-NCM cathode composites for solid-state batteries studied by transmission electron microscopy. Matter.

[32] Shrestha S., Kim J., Jeong J., Lee H.J., Kim S.C., Hah H.J., Song M.S., Oh K., Lee S.H. (2022). Effect of Polyacrylonitrile Surface Coating on Electrochemical Performance of LiNi_0.8_Mn_0.1_Co_0.1_O_2_ in All Solid-State Batteries. J. Electrochem. Soc..

[33] Wu M., Song H., Zhou X., Qin L., Fan X., Wang H. (2024). Li_1.4_Al_0.4_Ti_1.6_(PO_4_)_3_ coating surface modification enables improved electrochemical performance of LiNi_0.83_Co_0.11_Mn_0.06_O_2_ cathode. Mater. Today Commun..

[34] Liu Y., Shao Z., Chen Y., Yu T., Dong Z., Yang H., Yu Y.-G. (2024). Stabilizing Phase Transition in Ni Rich Layered Oxide Cathode Material by Nanoscale Nasicon Polyanion Groups. https://ssrn.com/abstract=4809101.

[35] Zhang M., Zhang P., Wen W.D., Wang H.W., He B.B., Gong Y.S., Jin J., Wang R. (2022). Enhanced Electrochemical Performance of LiNi_1/3_Co_1/3_Mn_1/3_O_2_ at a High Cut-Off Voltage of 4.6 V by Li_1.3_Al_0.3_Ti_1.7(_PO_4_)_3_ Coating. Coatings.

[36] Yen P.Y., Lee M.L., Gregory D.H. (2020). and Liu, W.R. Optimization of sintering process on Li_1+_*_x_*Al*_x_*Ti_2-_*_x_*(PO_4_)_3_ solid electrolytes for all-solid-state Lithium-ion batteries. Ceram. Int..

[37] Xiao Y.H., Miara L.J., Wang Y., Ceder G. (2019). Computational Screening of Cathode Coatings for Solid-State Batteries. Joule.

[38] Benabed Y., Rioux M., Rousselot S., Hautier G., Dollé M. (2021). Assessing the Electrochemical Stability Window of NASICON-Type Solid Electrolytes. Front. Energy Res..

[39] Chen F., Zhu X.Q., Dai W.L., Yao C.C., Qian J.C., Chen Z.G., Liu C.B. (2022). Optimized Ni-rich LiNi_0.83_Co_0.06_Mn_0.06_Al_0.05_O_2_ cathode material with a Li_1.3_Al_0.3_Ti_1.7_(PO_4_)_3_ fast ion conductor coating for Lithium-ion batteries. J. Alloys Compd..

[40] Divakaran A.M., Minakshi M., Bahri P.A., Paul S., Kumari P., Divakaran A.M., Manjunatha K.N. (2021). Rational design on materials for developing next generation lithium-ion secondary battery. Prog. Solid State Chem..

[41] Subashini C., Sivasubramanian R., Sundaram M.M., Priyadharsini N. (2025). The evolution of allotropic forms of Na_2_CoP_2_O_7_ electrode and its role in future hybrid energy storage. J. Energy Storage.

