# Supplementary material for: Interface Engineering of NCMA Cathodes with LATP Coatings for High-Performance Solid-State Lithium Batteries
Source: Nanomaterials (Basel). 2025 Jul 8;15(14):1057. doi: 10.3390/nano15141057 (PMC12300180; doi:10.3390/nano15141057)
Supplement: Supplementary file 1 [file nanomaterials-15-01057-s001.zip › nanomaterials-3737272-supplementary.pdf]

Supplementary Materials

Interface Engineering of NCMA Cathodes with LATP Coatings for High-Performance Solid-State Lithium Batteries

Shih-Ping Cho [1], Muhammad Usman Hameed [2], Chien-Te Hsieh [3,4,*] and Wei-Ren Liu [1,5,6,*]

[1] Department of Chemical Engineering, R&D Center for Membrane Technology, Chung Yuan Christian University, 200 Chung Pei Road, Chungli District, Taoyuan 32023, Taiwan
[2] Department of Chemistry, University of Poonch Rawalakot, Azad Kashmir 12350, Pakistan
[3] Department of Chemical Engineering and Materials Science, Yuan Ze University, Taoyuan 32003, Taiwan
[4] Department of Mechanical, Aerospace, and Biomedical Engineering, University of Tennessee, Knoxville, TN 37996, USA
[5] Hierarchical Green-Energy Materials (Hi-GEM) Research Center, National Cheng Kung University, 1 University Road, Tainan 70101, Taiwan
[6] Department of Chemical Engineering, Faculty of Engineering, Chulalongkorn University, Bangkok 10330, Thailand

* Correspondence: cthsieh@saturn.yzu.edu.tw (C.-T.H.); wrliu1203@gmail.com (W.-R.L.); Tel.: +886-3-265-4140 (W.-R.L.); Fax:+886-3-265-4199 (W.-R.L.)

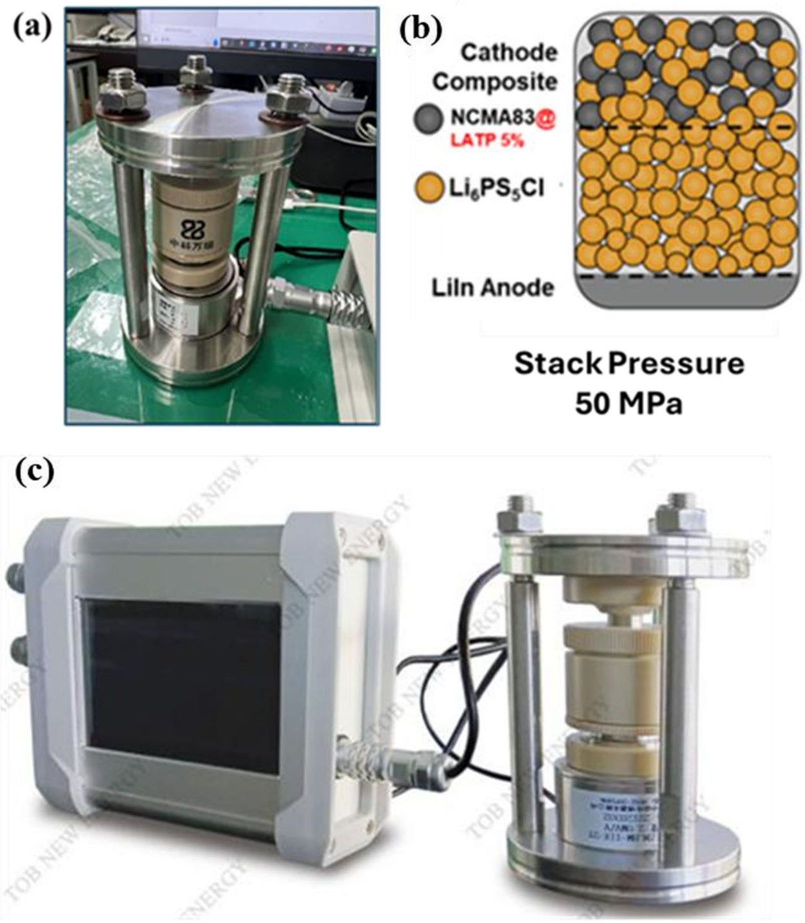

Figure S1. (a) and (c) Stack cell; (b) Composition of all solid-state battery.

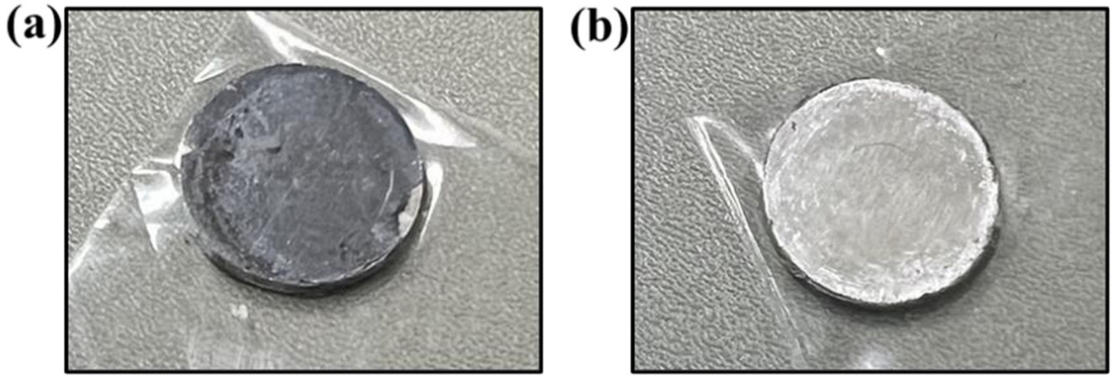

Figure S2. (a) Cathode side of cell pellet, (b) LiIn metal anode side of cell pellet.

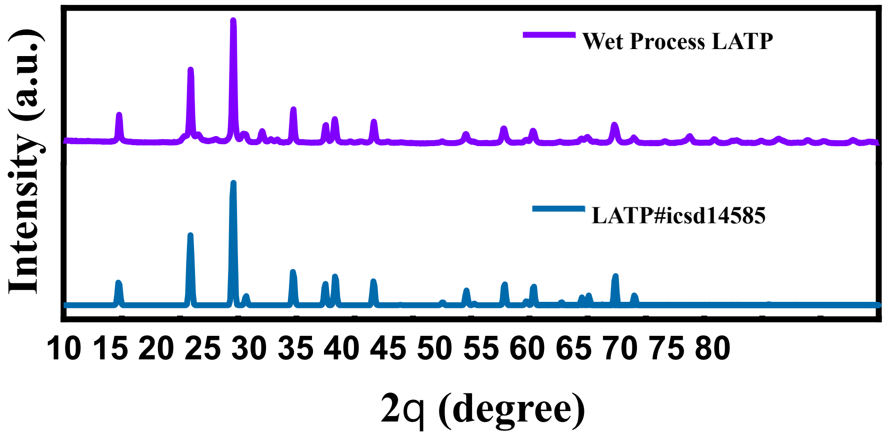

Figure S3. XRD patterns of as-synthesized LATP (namely Wet LATP) and its standard pattern taken from ICSD 14585**.**

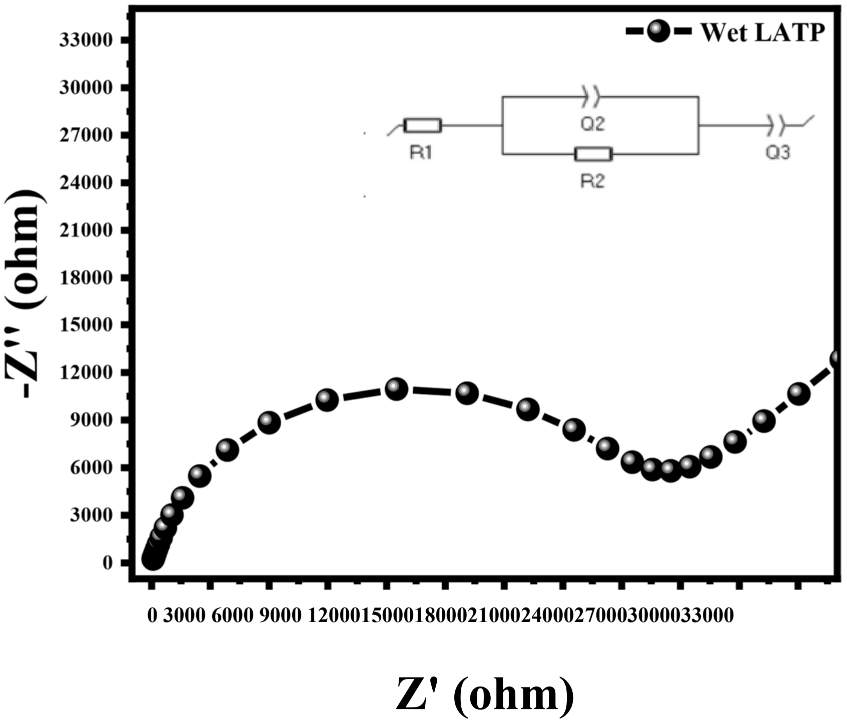

Figure S4. EIS of as-synthesized LATP.

Table S1. Ionic conductivity of LATP.

Samples	L (μm)	Area (cm^2)	R_1 (Ω)	R_2 (Ω)	R_{total} (Ω)	Ionic conductivity (S/cm)
LATP	360	0.785	0.5	23442	23442	1.21*10^{-5}

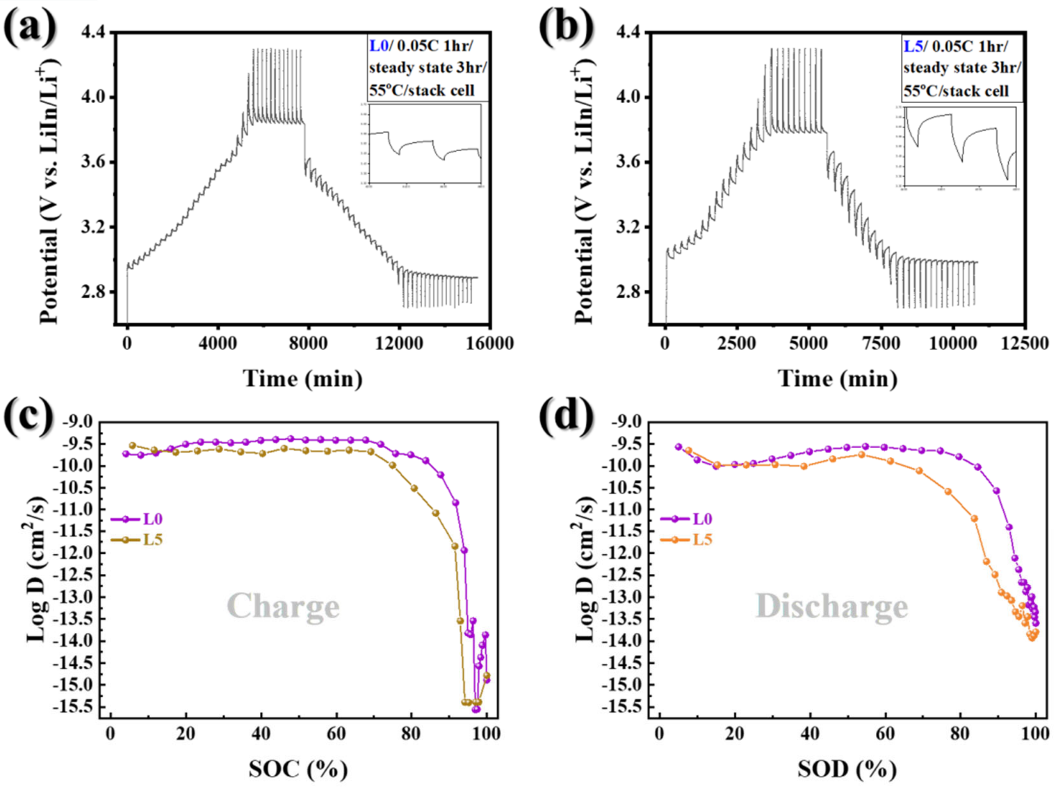

Figure S5. (a), (b), (c) and (d): GITT for pristine cathode and 5%LATP coated cathode.

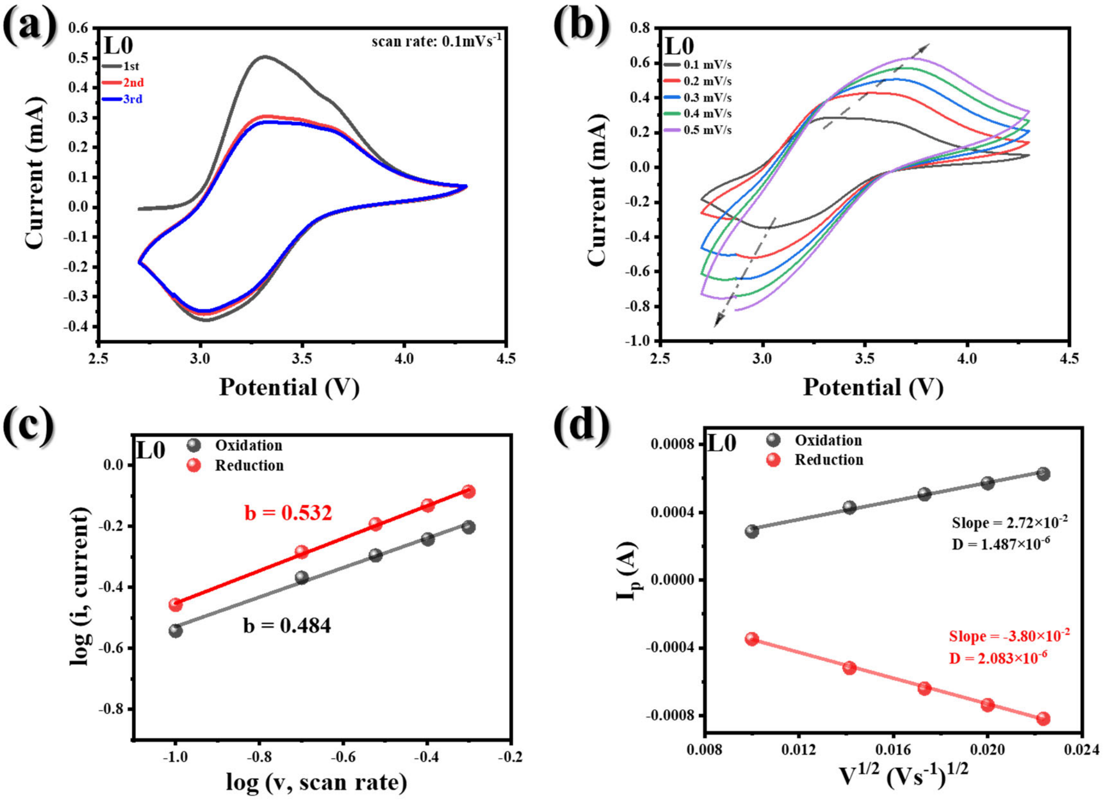

Figure S6. L0 (Pristine cathode) CV (a) the first 3 cycles, (b) different scan V; (c) the b value, and (d) D value.

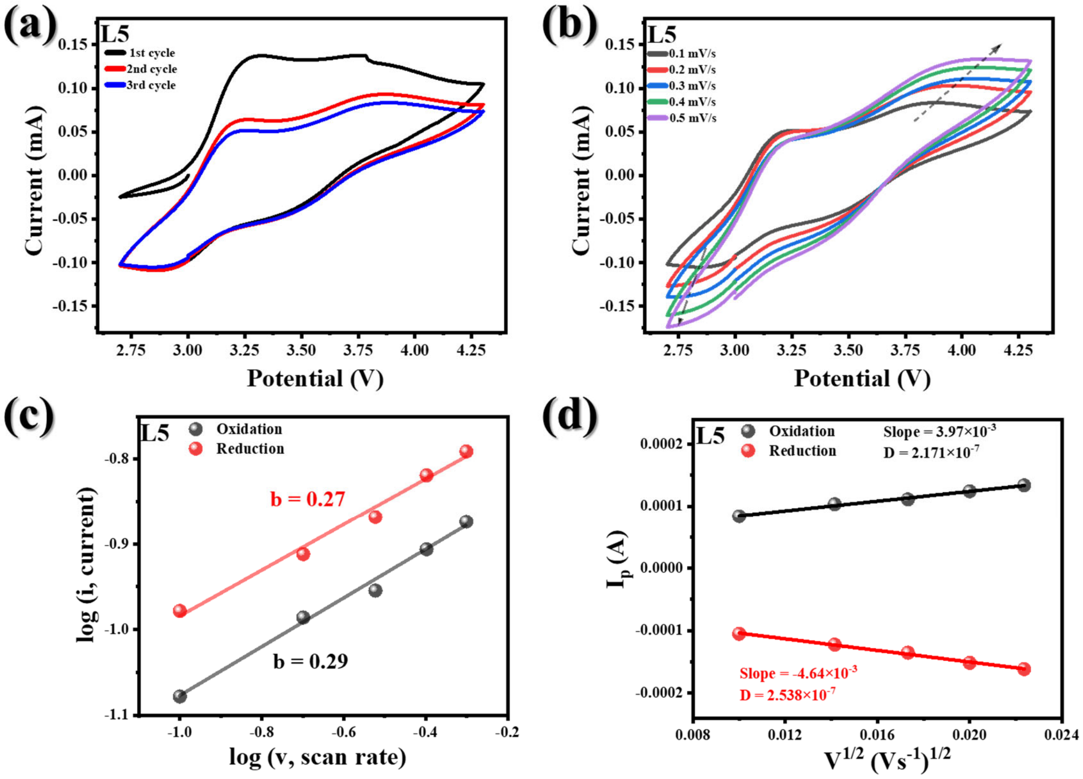

Figure S7. L5 (5% LATP-coated cathode) CV (a) the first 3 cycles, (b) different scan V; (c) the b value, and (d) D value.

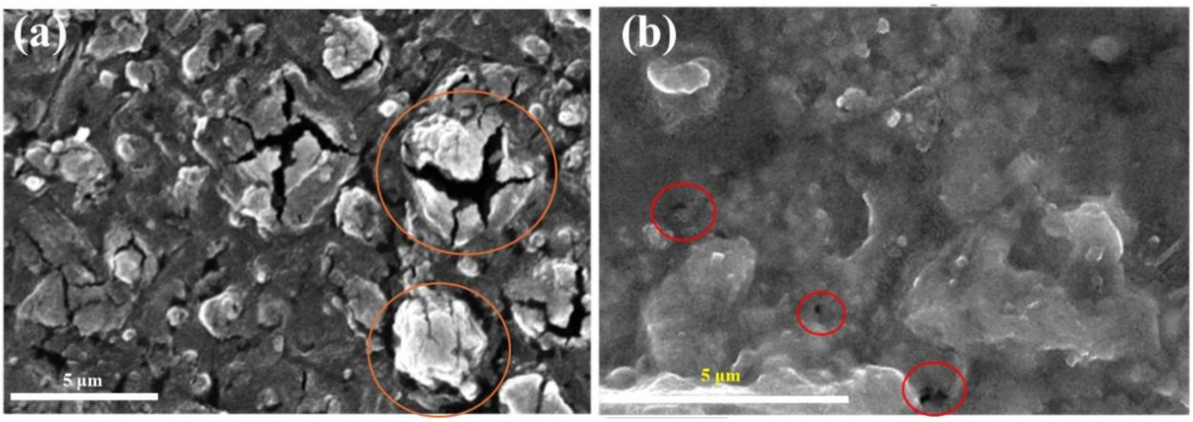

Figure S8. SEM images of (a) 83L0 and (b) 83L5 composite cathode after 50 charge-discharge cycles.
